# Clinical Characteristics of Early-Onset and Late-Onset Leigh Syndrome

**DOI:** 10.3389/fneur.2020.00267

**Published:** 2020-04-15

**Authors:** Chan-Mi Hong, Ji-Hoon Na, Soyoung Park, Young-Mock Lee

**Affiliations:** ^1^Departments of Pediatrics, Yonsei University College of Medicine, Seoul, South Korea; ^2^Department of Pediatrics, Soon Chun Hyang University Hospital and College of Medicine, Soonchunhyang University, Bucheon-si, South Korea

**Keywords:** Leigh syndrome, late onset, early onset, prognosis, pediatric, mitochondrial disease

## Abstract

**Background:** Leigh syndrome (LS) is the most common pediatric mitochondrial diseases caused by MRC defect. LS patients typically have onset age before 2 years old and have various clinical features. The purpose of this study was to evaluate the various characteristics between the group that were early onset and late onset patients.

**Methods:** The medical records of this study used records between 2006 and 2017 (*N* = 110). Clinical characteristics, diagnostic evaluations, and neuro image studying of LS were reviewed in our study. We statistically analyzed data from patients diagnosed with LS at our hospital by using subgroup analysis was performed to divide patients according to the onset age.

**Results:** Among the patients, 89 patients (80.9%) had the onset age before 2 years old, and 21 patents (19.1%) had onset age after 2 years old. In subgroup analysis first clinical presentation age, diagnosis age and several onset symptoms in the clinical characteristics were statistically significant. Early onset age group showed delayed development and late onset age group showed motor weakness and ataxia. However, Diagnostics evaluation and MRI findings showed no significant differences. The clinical status monitored during the last visit showed statistically significant differences in the clinical severity. In the early onset age group clinical status was more severe than late onset age group.

**Conclusions:** Although the onset of Leigh syndrome is known to be under 2 years, there are many late onset cases were existed more than expected. Early onset LS patients have poor prognosis compare with late onset LS patients. Therefore, the specific phenotype according to the age of onset should be well-observed. Onset of LS is important in predicting clinical severity or prognosis, and it is necessary to provide individualized treatment or follow-up protocols for each patient.

## Introduction

Leigh syndrome (LS) is an early-onset progressive neurodegenerative disease representing the most common pediatric clinical presentation of mitochondrial disease (1–3). LS was named after Denis Leigh, the first man to describe this rare neuropathology of infants and young children, which caused death in the affected patients. LS is known to affect about 1 in 40,000 births (4–7).

Although LS is associated with considerable clinical and genetic heterogeneity, the characteristic neuropathological features are remarkably consistent. Patients with LS most commonly manifest a progressive decline of central nervous system function due to focal, necrotizing lesions of the basal ganglia, diencephalon, cerebellum, or brainstem (5). The clinical hallmarks of LS include psychomotor delay or regression, weakness, hypotonia, truncal ataxia, intention tremor, and lactosidosis of the blood, cerebrospinal fluid (CSF), or urine (8).

The clinical manifestations of LS usually persist for up to 2 years after the initial development. LS patients are typically described to have the onset time before 2 years of age along with various clinical features. The disease course is associated with episodic neurodegeneration, often terminating with death at the age of 3 years (4, 6, 7, 9–11). Although adult-onset LS has been reported, it is very rare (12, 13). The purpose of this study was to evaluate the clinical features, and laboratory and image findings of LS by comparing these findings in patients with early-onset and late-onset LS.

## Materials and Methods

### Patients Group

This study was a retrospective analysis of patients diagnosed with LS and followed up at Gangnam Severance Hospital from January 2006 to January 2017. Patients who fulfilled the following criteria: (1) progressive neurological disease with motor and intellectual developmental delay; (2) signs and symptoms of brainstem and/or basal ganglia disease; (3) raised lactate levels in the blood and/or CSF, and (4) one or more of the following: (a) characteristic features of LS on neuroradioimaging [symmetrical hypodensities in the basal ganglia on computed tomography (CT) or hyperintense lesions on T2-weighted magnetic resonance imaging (MRI)], (b) typical neuropathological changes postmortem, or (c) typical neuropathology in a similarly affected sibling (14) were included.

The Institutional Review Board of Gangnam Severance Hospital in Seoul, Korea approved all our procedures. Informed consent was obtained from the parents, and all methods were performed in accordance with relevant guidelines and ethics board regulations.

### Data Collection Regarding Clinical Characteristics, Diagnostic Evaluation, Neuroimaging and Clinical Status at the Last Visit

Clinical data on the age at first clinical presentation, age at diagnosis, period from the first symptoms to the last visit, birth history, family history, nature of the first symptom, and organ involvement were collected.

Laboratory test results, including serum lactic acid levels and creatine kinase levels, were also obtained. The degree of serum lactic acidosis was defined as mild, moderate, or severe if the increase was <2, <3, or >3-fold higher than the normal reference values, respectively. Biochemical enzyme assay of the muscle tissue was also performed to evaluate the mitochondrial respiratory chain (MRC) complex enzyme activity. MRC complex enzyme defect was defined as a residual enzyme activity of <10% the reference value. Muscle biopsies were performed on the quadriceps muscle, and histologic examinations were performed using a light microscope and an electron microscope. Specific findings for mitochondrial diseases under a light microscope were defined as the presence of ragged red fibers (RRF) or abnormal staining. Abnormal mitochondrial morphology was defined under the electron microscope as pleoconia and megaconia. Data from genetic test of nuclear DNA (nDNA) and mitochondrial DNA (mtDNA) was collected. Brain MRI studies were also performed. The images used in this study were taken at the time of diagnosis of LS.

The clinical severity of LS in the patients was defined as: normal, ambulatory, and independent for daily activities; mild, ambulatory, or independent for daily activities; moderate, wheelchair-bound, or partially dependent for daily activities; and severe, bedridden, totally dependent for daily activities, or dead. At the last visit, data on whether they had undergone tracheostomy, home vent use, O_2_ dependence, and enteral tube feeding were also collected.

### Subgroup Analysis by Age at Onset

A total of 110 patients were recruited in this study. Patients were divided into two groups based on their age at LS onset. Age at LS onset was divided into onset age of later than the second year of life (late onset group) and onset age of before the second year of life (early-onset group). Clinical features, laboratory findings, and image findings were compared between the two groups.

### Statistical Analysis

All analyses were conducted using the Statistical Package for the Social Sciences (SPSS) version 22.0 (IBM Corp., Armonk, NY, USA). Descriptive statistics were used, including the median and range. Differences between subgroups were evaluated using the Mann-Whitney *U*-test (Wilcoxon rank sum test) and Fisher's exacts test. *P*-values of < 0.05 were considered statistically significant.

## Results

### Distribution of LS by Age at Onset

A total of 110 patients were diagnosed with LS. Among the patients, 89 patients (80.9%) had an onset age of before 2 years old, and 21 patents (19.1%) had an onset age of after 2 years old. In 89 patients with a typical onset age of <2 years old, 17 patients (15.5%) were between 1 and 2 years old and 72 patients (65.5%) were under 1 year of age.

### Patient Characteristics and Clinical Features

Of the 110 patients who were diagnosed with LS, 51 (46.4%) patients were male and 59 (53.6%) were female. The median age at the first clinical presentation was 9 months (range 0–186 months). The median age at the diagnosis of LS was 25months (range 3–244 months). The time interval between the first clinical presentation and the diagnosis of Leigh syndrome was 13 months (range 0–173 months). The time duration from LS diagnosis to the last outpatient clinic visit was 109 months (range 4–290 months). The median duration of follow-up from symptom onset to the last visit was 83 months (range 0–211 months). Regarding birth history, a higher number of the infants were admitted in the neonatal intensive care unit (13 patients, 11.8%). Of the 16 patients who had a family history, 13 patients (11.8%) had mitochondrial disease history and 3 patients (2.7%) had a non-specific neurological disease history. In the subgroup analysis, the age at onset was divided into onset age of over 2 years old and onset age of under 2 years, and the age at first clinical presentation and the age at LS diagnosis were statistically significant (*P* < 0.05)

The presenting symptom at disease onset varied, with delayed development (55 patients, 45.5%) as the most common, followed by seizures (24 patients, 21.6%), motor weakness (11 patients, 9.5%), drowsiness (7 patients, 6.3%), ataxia (5 patients, 4.5%), muscle atrophy (3 patients, 2.7%), and visual disturbance (2 patients, 1.8%). There were three significant differences in presenting symptoms between the early-onset and late-onset groups. Delayed development was significantly higher in the early-onset group than in the late-onset group. Motor weakness and ataxia were significantly higher in the late-onset group than in the early-onset group. Among those 24 patients who showed seizure as the presenting symptom at disease onset, we found that 11 patients (45.8%) had a focal seizure locus while 8 (33.3%) had generalized seizure semiology. Complex partial seizure was the most common type of focal seizure observed. As for generalized seizure types, myoclonic seizure, tonic seizure, atonic seizure, and tonic-clinic seizure were observed with relatively even prevalence among patients. In addition, two patients showed partial seizure with secondary generalization and three patients had unclassified epileptic seizures. There was no significant difference in seizure types between the early-onset and late-onset groups.

All the patients experienced involvement of the central nervous system, followed by the muscular systems, gastrointestinal and respiratory systems, and several organs. Among the organ involvements, involvement of the muscular systems, such as myopathy, creatine kinase level elevation, and positive muscle biopsy findings, was significantly higher in the late-onset group (Table 1).

**Table 1:** Clinical characteristics of Leigh syndrome.

	**Total**		**Subgroup by age at onset**				***P-*value**
			**Age** **≤** **2yr (*****n*** **=** **89)**		**Age** **>** **2yr (*****n*** **=** **21)**		
**Sex**							0.228
Male	51	46.4 (%)	44	49.4 (%)	7	33.3 (%)	
Female	59	53.6 (%)	45	50.6 (%)	4,514	66.7 (%)	
*Age at the first clinical presentation (month)	9	0−186	7	0–24	44	25–186	<0.001
*Age at the diagnosis of LS (month)	25	3−244	21	3–185	60	25–244	<0.001
*The interval between the first clinical presentation and diagnosis of LS (month)	13	0−173	13	0–153	12	0–173	0.698
*The duration from LS diagnosis to the last clinic visit (month)	109	4−290	109	4–281	117	22–290	0.822
*The duration of follow-up from symptom onset to the last clinic visit (month)	83	0−221	82	0–221	83	0–147	0.976
**Birth history**							
Prematurity	4	3.6 (%)	4	4.5 (%)	0	0.0 (%)	0.423
Intrauterine growth retardation	5	4.5 (%)	5	5.6 (%)	0	0.0 (%)	0.126
Perinatal asphyxia	7	6.4 (%)	4	4.5 (%)	3	14.3 (%)	0.241
Neonatal intensive care unit history	13	11.8 (%)	12	13.5 (%)	1	4.8 (%)	0.339
**Family history**	16	14.5 (%)	11	12.4 (%)	5	23.8 (%)	0.406
Mitochondrial disease history	13	11.8 (%)	9	10.1 (%)	4	19.0 (%)	
Non-specific neurological disease history	3	2.7 (%)	2	2.2 (%)	1	4.8 (%)	
**Presenting symptom at disease onset**							
Delayed development	55	49.5 (%)	50	56.2 (%)	5	23.8 (%)	0.008
Seizure	24	21.6 (%)	20	22.5 (%)	4	19.0 (%)	0.495
Motor weakness	11	9.9 (%)	6	6.7 (%)	5	23.8 (%)	0.019
Drowsiness	7	6.3 (%)	5	5.6 (%)	2	9.5 (%)	0.400
Ataxia	5	4.5 (%)	2	2.2 (%)	3	14.3 (%)	0.047
Muscle atrophy	3	2.7 (%)	3	3.4 (%)	0	0.0 (%)	0.526
Visual disturbance	2	1.8 (%)	1	1.1 (%)	1	4.8 (%)	0.347
Ear disorder	1	0.9 (%)	1	1.1 (%)	0	0.0 (%)	0.809
Behavior change	1	0.9 (%)	0	0.0 (%)	1	4.8 (%)	0.191
Ptosis	1	0.9 (%)	1	1.1 (%)	0	0.0 (%)	0.809
**Organ involvement**							
Central nervous system	110	100 (%)	89	100.0 (%)	21	100.0 (%)	–
Muscular	52	47.3 (%)	38	42.7 (%)	14	66.7 (%)	0.048
Gastrointestinal	41	37.3 (%)	35	39.3 (%)	6	28.6 (%)	0.359
Respiratory	33	30.0 (%)	29	32.6 (%)	4	19.0 (%)	0.171
Ophthalmic	17	15.5 (%)	15	16.9 (%)	2	9.5 (%)	0.323
Cardiologic	9	8.2 (%)	9	10.1 (%)	0	0.0 (%)	0.137
Renal	8	7.3 (%)	8	9.0 (%)	0	0.0 (%)	0.172
Auditory	6	5.5 (%)	4	4.5 (%)	2	9.5 (%)	0.322
Hematologic	1	0.9 (%)	0	0.0 (%)	0	0.0 (%)	–
Endocrinologic	1	0.9 (%)	1	1.1 (%)	0	0.0 (%)	0.809

* *Continuous variables are descripted by medians and ranges*.

*Other variables are descripted by number of patients and percentage (%) in each group*.

### Diagnostic Evaluation of Leigh Syndrome

The median serum lactate level at the time of diagnosis was 2.6 (range 0.9–20.7), and at the time of the last visit, it was 2.0 (range 0.6–14.0). Serum lactic acid level was increased in 64 patients (58.7%). Serum levels of lactic acid were found to be mildly increased, moderately increased, and severely increased in 26.6, 24.8, and 7.3% of patients, respectively. In most cases, serum creatine kinase levels were normal both at the time of the diagnosis and at the last visit.

Biochemical evaluation of MRC complex enzyme function in the muscle tissue of 92 patients (no biochemical evaluation results were found for 18 patients) showed defects in MRC complex I and MRC complex IV in 53 patients (57.6%) and 34 patients (37.0%), respectively. Muscle biopsy, performed for 100 of the 110 patients, showed abnormal changes under a light microscope in 25 patients (25%), including nonspecific findings (3 patients), specific mitochondrial disease (22 patients), such as RRF and abnormal staining. Abnormal findings under an electron microscope were observed in 72 patients (72%); these included pleoconia (37 patients) and megaconia (35 patients).

There was no significant difference between the subgroups in lactic acidosis and elevation of serum creatine kinase levels. In addition, no significant difference in MRC complex enzyme assay and muscle biopsy findings was observed in the subgroup analysis (Table 2).

**Table 2:** Diagnostic evaluation of Leigh syndrome.

	**Total**		**Subgroup by age at onset**				***P-*value**
			**Age** **≤** **2yr (*****n*** **=** **89)**		**Age** **>** **2yr (*****n*** **=** **21)**		
**Lactate (mmol/L)**							
*At the time of diagnosis	2.6	0.9–20.7	2.6	0.9–20.7	2.4	1.2–7.0	0.563
*Last visit	2.0	0.6–14.0	2.1	0.8–14.0	1.9	0.6–4.3	0.086
**Lactate/pyruvate ratio**							
*At the time of diagnosis	16.8	4.8–260.0	17.0	4.8–260.0	15.7	7.1–40.0	0.396
*Last visit	16.1	4.8–176.9	16.8	4.8–68.6	15.7	5.0–176.9	0.142
**Lactic acidosis (*****n*** **=** **109)**			(*n* = 89)		(*n* = 20)		
Normal	45	41.3 (%)	36	40.4 (%)	9	45.0 (%)	
Mild increased (≥2-fold)	29	26.6 (%)	22	24.7 (%)	7	35.0 (%)	0.193
Moderate increased (≥3-fold)	27	24.8 (%)	24	27.0 (%)	3	15.0 (%)	
Severe increased (≥4-fold)	8	7.3 (%)	7	7.9 (%)	1	5.0 (%)	
**Creatine kinase (mmol/L)**							
*At the time of diagnosis	101	15−1, 257	99	15–1,257	109	22–409	0.948
Normal	75	68.2 (%)	58	65.2 (%)	56	62.9 (%)	
Mild increased (<2-fold)	29	26.4 (%)	26	29.2 (%)	25	28.1 (%)	0.995
Severe increased (≥2-fold)	6	5.5 (%)	5	5.6 (%)	8	9.0 (%)	
*Last visit	113	7–873	117	7–873	105	27–595	0.550
Normal	69	62.7 (%)	17	81.0 (%)	13	61.9 (%)	
Mild increased (<2-fold)	31	28.2 (%)	3	14.3 (%)	6	28.6 (%)	0.353
Severe increased (≥2-fold)	10	9.1 (%)	1	4.8 (%)	2	9.5 (%)	
**Plasma amino acid (*****n*** **=** **88)**							
*Alanine	389	102–1,028	390	158–1,028	378	102–652	0.303
Normal	52	59.1 (%)	41	57.7 (%)	11	64.7 (%)	
Mild increased (≥2-fold)	31	35.2 (%)	25	35.2 (%)	6	35.3 (%)	0.721
Severe increased (≥4-fold)	5	5.7 (%)	5	7.0 (%)	0	0.0 (%)	
*Glycine	213	98–8,010	208	108–8,010	217	98–355	0.763
Normal	87	97.8 (%)	70	97.2 (%)	17	100.0 (%)	
Abnormal (≥2-fold)	2	2.2 (%)	2	2.8 (%)	0	0.0 (%)	0.785
*Proline	173	0–518	176	0–518	133	0–232	0.021
Normal	82	92.1 (%)	65	90.3 (%)	17	100.0 (%)	
Abnormal (≥2-fold)	7	7.9 (%)	7	9.7 (%)	0	0.0 (%)	0.408
*Threonine	116	38–762	117	38–762	101	43–177	0.031
Normal	94	85.5 (%)	78	92.9 (%)	16	94.1 (%)	0.128
Abnormal (≥2-fold)	7	6.4 (%)	6	7.1 (%)	1	5.9 (%)	
**Urine organic acid (*****n*** **=** **71)**			(*n* = 56)		(*n* = 15)		0.819
Abnormal finding	36	50.7 (%)	28	50 (%)	8	53.3 (%)	
**MRC complex enzyme assay (*****n*** **=** **92)**			(*n* = 75)		(*n* = 17)		0.888
MRC complex I defect	53	57.6 (%)	42	47.2 (%)	11	64.7 (%)	
MRC complex IV defect	34	37.0 (%)	29	32.6 (%)	5	29.4 (%)	
**Muscle biopsy obtained**			(*n* = 80)		(*n* = 20)		
Light microscopic changes (+)							0.296
Specific findings for mitochondrial diseases	22	22 (%)	15	18.8 (%)	7	35.0 (%)	
Non-specific findings	3	3 (%)	3	3.8 (%)	0	0.0 (%)	
Electron microscopic changes (+)							
Pleoconia	37	37 (%)	26	32.5 (%)	11	55.0 (%)	0.123
Megaconia	35	35 (%)	25	31.3 (%)	10	50.0 (%)	0.207

* *Continuous variables are descripted by medians and ranges*.

*Other variables are descripted by number of patients and percentage (%) in each group*.

In our study, genetic test of whole mtDNA and nDNA was not performed in every patient. Out of 110 patients with Leigh syndrome who met the clinical criteria, three patients showed nDNA mutation and all had *SURF1* gene mutation. The mtDNA mutation was confirmed in 22 patients (Supplementary Tables 1, 2).

### MRI Findings in Leigh Syndrome

MRI of the brain revealed several abnormal findings in the patients, including atrophy or abnormal signals in different areas of the brain. Basal ganglia (104 patients, 94.5%) was the most common abnormal MRI findings, followed by cerebral atrophy (64 patients, 58.2%), and brainstem (45 patients, 40.9%), and white matter signal abnormality (42 patients, 38.2%). There was no significant difference between the subgroup in MRI findings (Table 3).

**Table 3:** Magnetic resonance imaging findings.

	**Total**		**Subgroup by age at onset**				***P-*value**
			**Age** **≤** **2yr (*****n*** **=** **89)**		**Age** **>** **2yr (*****n*** **=** **21)**		
**Basal ganglia**	104	94.5 (%)	84	94.4 (%)	20	95.2 (%)	0.678
**Thalamus**	38	34.5 (%)	33	37.1 (%)	5	23.8 (%)	0.071
**Brainstem**	45	40.9 (%)					
Midbrain	44	40.0 (%)	35	39.3 (%)	9	42.9 (%)	0.766
Pons	27	24.5 (%)	22	24.7 (%)	5	23.8 (%)	0.931
Medulla	30	27.3 (%)	23	25.8 (%)	7	33.3 (%)	0.488
**Number of brain stem parts involved**							0.067
0	62	56.4 (%)	51	57.3 (%)	11	52.4 (%)	
1	17	15.5 (%)	15	16.9 (%)	2	9.5 (%)	
2	10	9.1 (%)	5	5.6 (%)	5	23.8 (%)	
3	21	19.1 (%)	18	20.2 (%)	3	14.3 (%)	
**Cerebellum**	41	37.3 (%)	35	39.3 (%)	6	28.6 (%)	0.359
**Cerebral atrophy**	64	58.2 (%)					0.332
Mild	49	44.5 (%)	40	44.9 (%)	9	42.9 (%)	
Severe	15	13.6(%)	14	15.7 (%)	1	4.8 (%)	
**Cortex signal abnormality**	33	30.0 (%)	28	31.5 (%)	5	23.8 (%)	0.491
**White matter signal abnormality**	42	38.2 (%)					0.524
Mild	40	36.4 (%)	34	38.2 (%)	6	28.6 (%)	
Severe	2	1.82 (%)	2	2.2 (%)	0	0.0 (%)	

### Clinical Status at Last Visit

At the last outpatient clinic visit, 9 patients (8.2%) showed a mild clinical severity (ambulatory and/or independent for daily activities), 32 patients (29.1%) showed moderate clinical severity (wheelchair-bound and/or partially dependent for daily activities, able to express and understand direction), 58 patients (52.7%) showed critical clinical severity (bedridden, total dependence for daily activities), and the other patients were dead (11 patients, 10%). In addition, 27 patients underwent tracheostomy, 25 patients utilized home ventilator, 23 patients showed O_2_ dependence, and 38 patients were on enteral tube feeding.

By dividing the patients into subgroups according to onset age, we found differences in clinical severity at the last visit to be statistically significant. The clinical status of the early-onset group was more severe (59.6%) than that of the late-onset group. Therefore, the early- onset group had a poor prognosis compared to the late-onset group. There was no significant difference between the subgroups in previous tracheostomy, home vent use, O_2_ dependence, and enteral tube feedings (Table 4).

**Table 4:** Clinical status at the last visit.

	**Total**		**Subgroup by age at onset**				***P-*value**
			**Age** **≤** **2yr (*****n*** **=** **89)**		**Age** **>** **2yr (*****n*** **=** **21)**		
**Clinical severity**							0.004
Mild	9	8.2 (%)	5	5.6 (%)	4	19.0 (%)	
Moderate	32	29.1 (%)	21	23.6 (%)	11	52.4 (%)	
Severe	58	52.7 (%)	53	59.6 (%)	5	23.8 (%)	
Dead	11	10.0 (%)	10	11.2 (%)	1	4.8 (%)	
**Regression**	73	66.4 (%)	59	66.3 (%)	14	66.7 (%)	0.974
**Tracheostomy**							0.177
Yes	27	24.5 (%)	24	26.9 (%)	3	14.3 (%)	
No	83	75.5 (%)	65	73.0 (%)	18	85.7 (%)	
**Home ventilator use**							0.236
Yes	25	22.7 (%)	22	24.7 (%)	3	14.3 (%)	
No	85	77.3 (%)	67	75.3 (%)	18	85.7 (%)	
**O**_**2**_ **dependence**							0.453
Continuous	6	5.5 (%)	6	6.7 (%)	0	0.0 (%)	
Temporary	17	15.5 (%)	14	15.7 (%)	3	14.3 (%)	
No	87	79.1 (%)	69	77.5 (%)	18	85.7 (%)	
**Enteral tube feeding**							0.155
Temporary tube feeding	15	38.2 (%)	13	14.6 (%)	2	9.5 (%)	
Continuous tube feeding	4	38.2 (%)	4	4.5 (%)	0	0.0 (%)	
Gastrostomy feeding	19	17.3 (%)	18	20.2 (%)	1	4.8 (%)	

## Discussion

LS is traditionally considered to be a disease of infancy and early childhood that occurs before the age of 2 years (4, 15–17). More than 50% of cases are observed in patients within one year of age, usually six months old. Late-onset LS is rare worldwide (18). Karin et al. described 25 patients with LS, and all 25 patients had age onset younger than 2 years (16). Yang et al. described 65 patients with LS, and the age of LS onset in 59 patients was <1 year (19). In our study, the onset age ranged from <1 to 187 months. There are many criteria to classify age of onset, and clear criteria have not yet been established. Therefore, we divided early onset and late onset based on age of 2 years. Under 2 years old age onset were dominant in 80.9%.

According to previous reports, typical signs and symptoms may appear in late-onset LS (20). In late-onset Leigh syndrome, there was intellectual decline and vertical gaze paralysis, headache, memory loss, and visual hallucinations (21, 22). They were based on a small number of case reports. Generally, Leigh syndrome in adulthood is rare (20). Our results showed that delayed development, motor weakness, and ataxia among the presenting symptoms at disease onset were statistically significant. However, the time interval from the first clinical presentation to the diagnosis of LS, follow-up duration, birth history, family history, and organ involvement were not statistically significant. Therefore, Leigh syndrome should be considered when symptoms related to gross motor function such as delayed development, motor weakness, and ataxia are observed. In particular, these symptoms can be classified according to the age of onset. There is a frequency of difference between the specific symptoms in early onset and late onset patients. Delayed development is important in cases of early age of onset, and motor weakness or ataxia is important in cases of late age of onset. Consequently, mitochondrial disease should be suspected if the initial symptoms such as delayed development, motor weakness, and ataxia and regression are detected. Seizure and epilepsy in children with early-onset mitochondrial disease may be a presenting or a prominent symptom in a multisystemic clinical presentation. However, a higher prevalence of Leigh syndrome was detected in non-epileptic patients (23, 24). Though seizures took the place of the second most common presenting symptom at disease onset in our study, there was no case of severe condition as status epilepticus. The most frequent causes of mitochondrial status epilepticus are known as mitochondrial encephalomyopathy with lactic acidosis and stroke-like episodes (MELAS) and myoclonus epilepsy with ragged-red fibers (MERRF) (25).

LS is typically characterized by the involvement of the brainstem and/or basal ganglia (4, 26). Other areas of frequent involvement include the thalamus, cerebellum, and spinal cord (7, 14). Similar to previous studies, brainstem, basal ganglia, and thalamus involvement were more common in our results, with basal ganglia involvement being the most common. There was no significant difference between MRI findings according to onset age. Therefore, it is clinically meaningful to compare the interval changes in neuroimaging by individual follow up rather than MRI findings according to onset age. Although the consensus on when to repeat brain MRI in LS has not reached a definite conclusion yet, it is important to follow up MRI findings at regular intervals to compare changes.

The disease course is associated with episodic neurodegeneration, often ending with death until the age of 5 years (27). However, according to our results, the median time from the first symptom onset to the last visit was 83 months, which is longer than what was observed in previous studies. No causative treatment is available for Leigh disease because no treatment can reverse the sustained damage. Thus, the goal of each treatment are to reduce the expected deterioration rate and to alleviate symptoms such as respiratory disturbances and tube feeding. The reason for the prolongation of patient's survival period to 5 years or more is considered to be due to multidisciplinary treatment and supportive care, including serial monitoring and the use of various medication at LS diagnosis. The type of medication used supplements RC components like coenzyme Q, administers artificial electron acceptors like vitamin C and K, and administers metabolites and cofactors like carnitine, thiamin, and riboflavin (28). The clinical severity of LS in the early-onset group was worse than in the late onset group, and in the early-onset group, the proportion of patients with critical clinical severity or those who died was 70.8%, whereas in the late-onset group, 71.4% of patients had mild or moderate clinical severity. A difference in prognosis was shown according to onset age. Although there are reports of both early and late-onset LS, there is no comparative study of their prognosis. Therefore, it is necessary to compare their prognosis according to the age at onset.

Our study is a retrospective study with limited genetic diagnosis. A study conducted using retrospective design generally includes patients recruited over a long period making it practically impossible to perform genetic assessment in all patients and under the same condition. In our study, genetic tests for whole nDNA and mtDNA were not performed in all patients. Therefore, the number of patients with a confirmed mutation was too small to make statistically meaningful analysis. A study based on a large sample with full genetic assessment would be very helpful. Even a smaller cohort with genetic confirmation could be quite significant. However, the rarity of the disease makes it hard to find a study population big enough to provide generalized interpretations. That is also the reason for why studies on rare diseases are scarce when compared with those on common diseases. Despite the limitation of genetic diagnosis, standards of clinical diagnosis criteria, biochemical assay, and muscle pathology were applied very strictly in this study to make definite diagnosis. This was a large-scale study involving more than 100 patients in a single institution that meets previously established diagnostic criteria. Because LS is a rare disease, performing a prospective study has limitations because only a few cases will be available for research. However, we noted that early-onset and late-onset LS are similar in clinical phenotype and MRI finding, but their prognosis is quite different. In this study, we analyzed clinical phenotypes according to onset age. However, long-term studies involving a larger patient cohort with evaluation of the genotype of patients in each group could provide further prediction of the prognosis in LS. Individualized treatment is possible if we recognize the differences according to the onset age of LS. Therefore, the onset age of LS will ultimately be helpful for personalized treatment.

## Data Availability Statement

All datasets generated for this study are included in the article/supplementary material.

## Ethics Statement

The studies involving human participants were reviewed and approved by the Institutional Review Board of Gangnam Severance Hospital. Written informed consent to participate in this study was provided by the participants' legal guardian/next of kin.

## Author Contributions

Y-ML conceptualized and designed the study, coordinated and supervised data collection, and critically reviewed and revised the manuscript. C-MH, J-HN, and SP designed the data collection instruments, collected data, and carried out the initial analyses. C-MH drafted the initial manuscript and revised the manuscript. All authors approved the final manuscript as submitted and agree to be accountable for the content of the work.

### Conflict of Interest

The authors declare that the research was conducted in the absence of any commercial or financial relationships that could be construed as a potential conflict of interest.
