# Supplementary material for: Clinical Characteristics of Early-Onset and Late-Onset Leigh Syndrome
Source: Front Neurol. 2020 Apr 15;11:267. doi: 10.3389/fneur.2020.00267 (PMC7174756; doi:10.3389/fneur.2020.00267)
Supplement: Supplementary file 1 [file Table_1.pdf]

Table 1. Mitochondrial DNA(mtDNA) mutations in Leigh syndrome patients

mtDNA mutations	Total (N=22)
10191 T>C	4/22 (18.2%)
13513 G>A	4/23 (18.2%)
8993 T>G	4/22(18.2%)
8993 T>C	3/22 (13.6%)
9176 T>C	3/22 (13.6%)
3697 G>A	2/22 (9.1%)
10158 T>C	1/22 (4.5%)
11777 C>A	1/22 (4.5%)
