# Supplementary material for: Clinical Characteristics of Early-Onset and Late-Onset Leigh Syndrome
Source: Front Neurol. 2020 Apr 15;11:267. doi: 10.3389/fneur.2020.00267 (PMC7174756; doi:10.3389/fneur.2020.00267)
Supplement: Supplementary file 2 [file Table_2.pdf]

Table 2. Nuclear DNA(nDNA) mutations of *SURF1* gene in Leigh syndrome patients

Patient*	Allele 1	Allele 2
1	c.367_368delAG	c.844T>C
2	c.54+1G>T	c.809_826dup18
3	c.54+1G>T	c.831delC

* Genetic testing of nDNA was performed in 28 out of 110 patients with Leigh syndrome. Three patients (10.7%) showed nDNA mutation and all had *SURF1* gene mutation.
